# MR-PROTECT: Clinical feasibility of a prostate MRI-only radiotherapy treatment workflow and investigation of acceptance criteria

**DOI:** 10.1186/s13014-020-01513-7

**Published:** 2020-04-09

**Authors:** Emilia Persson, Christian Jamtheim Gustafsson, Petra Ambolt, Silke Engelholm, Sofie Ceberg, Sven Bäck, Lars E. Olsson, Adalsteinn Gunnlaugsson

**Affiliations:** 1 Radiation Physics, Department of Hematology, Oncology, and Radiation Physics, Skåne University Hospital, Klinikgatan 5, 221 85 Lund, Sweden; 2 Department of Translational Medicine, Medical Radiation Physics, Lund University, Inga-Marie Nilssons gata 49, 205 02 Malmö, Sweden; 3 Department of Medical Radiation Physics, Lund University, Barngatan 4, 222 85 Lund, Sweden

**Keywords:** MRI only, Prostate, Synthetic CT, Clinical implementation, Prospective, Acceptance criteria

## Abstract

**Background:**

Retrospective studies on MRI-only radiotherapy have been presented. Widespread clinical implementations of MRI-only workflows are however limited by the absence of guidelines. The MR-PROTECT trial presents an MRI-only radiotherapy workflow for prostate cancer using a new single sequence strategy. The workflow incorporated the commercial synthetic CT (sCT) generation software MriPlanner™ (Spectronic Medical, Helsingborg, Sweden). Feasibility of the workflow and limits for acceptance criteria were investigated for the suggested workflow with the aim to facilitate future clinical implementations.

**Methods:**

An MRI-only workflow including imaging, post imaging tasks, treatment plan creation, quality assurance and treatment delivery was created with questionnaires. All tasks were performed in a single MR-sequence geometry, eliminating image registrations. Prospective CT-quality assurance (QA) was performed prior treatment comparing the PTV mean dose between sCT and CT dose-distributions. Retrospective analysis of the MRI-only gold fiducial marker (GFM) identification, DVH- analysis, gamma evaluation and patient set-up verification using GFMs and cone beam CT were performed.

**Results:**

An MRI-only treatment was delivered to 39 out of 40 patients. The excluded patient was too large for the predefined imaging field-of-view. All tasks could successfully be performed for the treated patients. There was a maximum deviation of 1.2% in PTV mean dose was seen in the prospective CT-QA. Retrospective analysis showed a maximum deviation below 2% in the DVH-analysis after correction for rectal gas and gamma pass-rates above 98%. MRI-only patient set-up deviation was below 2 mm for all but one investigated case and a maximum of 2.2 mm deviation in the GFM-identification compared to CT.

**Conclusions:**

The MR-PROTECT trial shows the feasibility of an MRI-only prostate radiotherapy workflow. A major advantage with the presented workflow is the incorporation of a sCT-generation method with multi-vendor capability. The presented single sequence approach are easily adapted by other clinics and the general implementation procedure can be replicated. The dose deviation and the gamma pass-rate acceptance criteria earlier suggested was achievable, and these limits can thereby be confirmed. GFM-identification acceptance criteria are depending on the choice of identification method and slice thickness. Patient positioning strategies needs further investigations to establish acceptance criteria.

## Background

External beam radiotherapy (EBRT) is an important treatment to cure prostate cancer [1]. The prostate cancer EBRT workflow is commonly based on both Computed Tomography (CT) and Magnetic Resonance (MR) imaging [2]. The CT-images, with their electron density (ED) information, are used for treatment planning and the MR-images, with their superior soft tissue contrast, as a support for target and organs at risk (OAR) definition [3]. This dual-modality workflow demands an image registration between the MR- and CT-data sets. The image registration introduces a potential systematic spatial uncertainty of 1.7–2 mm, reported for patients with and without fiducial markers [4–7]. The improvement of future prostate EBRT regimens will most likely involve decreased number of treatment fractions with higher fractionation dose [8, 9] as well as steeper dose gradients between the target and organs at risk (OAR) [10]. This introduces the need for a more accurate dose delivery, without potential risk of image registration uncertainties. To accomplish this, a workflow with one image modality for both treatment planning and target delineation is needed. This will not only reduce potential registration uncertainties but also facilitate a more streamlined workflow for both patient and clinic. MR-imaging (MRI) makes an ideal foundation for this single-modality workflow – often referred to as MRI-only radiotherapy (RT) [11].

A conversion of the MR-data into a Hounsfield-unit (HU) representation is a prerequisite for dose-calculation in an MRI-only workflow, due to the lack of ED information in the MR-images. This HU-representation is often referred to as a synthetic CT, pseudo CT or substitute CT [12]. Numerous methods for prostate synthetic CT (sCT) generation have been presented and reviewed [12, 13]. Despite this, only a few studies about patient treatments with MRI-only workflows have been presented to date [14–18]. These workflows are based on either in-house methods [14, 15] or the commercially available sCT software MRCAT (Philips, Helsinki, Finland) [16–18].

Given that MRI-only is a new treatment approach, it is associated with several potential causes off failure of the workflow that need consideration. In the failure mode and effect analysis (FEMA) of a pelvis MRI-only workflow presented by Kim et al. [19] they identified key areas for risk mitigation in MRI-only. Generation of sCT was the major source of unique failure modes arising from either errors in acquired MR-data or caused by image processing required for sCT generation. Error mitigation could be accomplished with standardization of MR sequences, staff training and sCT quality assurance (QA). The results in this FEMA were based on a single center experience and was performed to foresee potential errors in a future implementation, rather than reporting results from previous clinical experience.

Clinical experience from running MRI-only has been reported along with failures in the workflows. Tennunen et al. [14] reported that 16 out of 200 patients in their study cohort needed additional CT-scanning. Three patients required conversion to the dual-modality workflow due to prostate artefacts in the MR-images caused by hip-implants. Problems were also reported related to fiducial marker identification, patient size and motion during MRI, but did not require a CT-based treatment. Tyagi et al. [17] reported that six patients out of 48 had to be converted to the dual-modality workflow due to hip-implants (*n* = 4) and obesity (*n* = 2). Difficulties with fiducial marker identification caused by motion artefacts in the MR-images were reported for two patients. This problem was overcome by additional Cone-Bream CT (CBCT)-appointments for verification, and the patients could subsequently undergo MRI-only treatment. Tyagi et al. suggested a CT-based QA-procedure to confirm correct GFM identification, which repeatedly has been reported as an obstacle in MRI-only [14, 17, 20, 21]. In contrast to the clinical workflows, which have been preceded by retrospective studies, a prospective implementation approach was recently investigated by Greer et al. for prostate MRI-only RT [15]. In their study, they used acceptance criteria for fiducial marker identification, iso-center dose and gamma evaluation with the CT as a prospective QA-tool. Using this method, they enabled and assessed safe implementation of MRI-only and treated all included patients (*n* = 25) with MRI-only RT. The study did not give any background on how the limits of the acceptance criteria were chosen.

A safe introduction of new treatments like MRI-only demands a thorough validation of each part of the workflow, using for instance the conventional CT for prospective QA. To the best of our knowledge, there is to date only one prospective implementation study presented for prostate MRI-only RT [15]. This approach, in comparison to earlier published clinical experiences, has higher success rate. This can hypothetically be due to the incorporation of acceptance criteria and a prospective QA approach. The prospective multi-center study presented by Greer et al. was based on an in-house sCT generation method, which may limit the possibility of a large scale implementation of their suggested method. Further, guidelines for clinical implementation of MRI-only today are missing in the literature, resulting in clinics having to struggle to adapt to this new treatment technique. Acceptance criteria could work as a powerful tool towards standardization of MRI-only and facilitate clinical implementations. More prospective studies are therefore required to lay the groundwork for the establishment of appropriate acceptance criteria, to be used in future implementations.

The aim of the MRI-only Prostate RadiOTherapy Excluding CT (MR-PROTECT) trial was to test the feasibility of a new proposed prostate MRI-only RT workflow using a single sequence strategy. A previously validated sCT generation method (MriPlanner™, Spectronic Medical AB, Helsingborg, Sweden) [22], was incorporated and used for treatments in the suggested workflow. A CT-QA was used for prospective investigation during implementation of the workflow. Fiducial marker identification, sCT dose-calculations and patient positioning were retrospectively investigated for the suggested workflow to investigate achievable limits for acceptance criteria.

## Methods

### Patient selection

Patients with localized prostate cancer were included in this study from March 2017 to May 2018. All patients were referred to RT of the prostate gland alone with patient set-up verification based on GFM, with MR- and CT-imaging as a part of their prescription. Patients with metallic implants in the pelvic area or any MR-contraindications were not considered for inclusion.

### MRI-only workflow

**Fig. 1:**
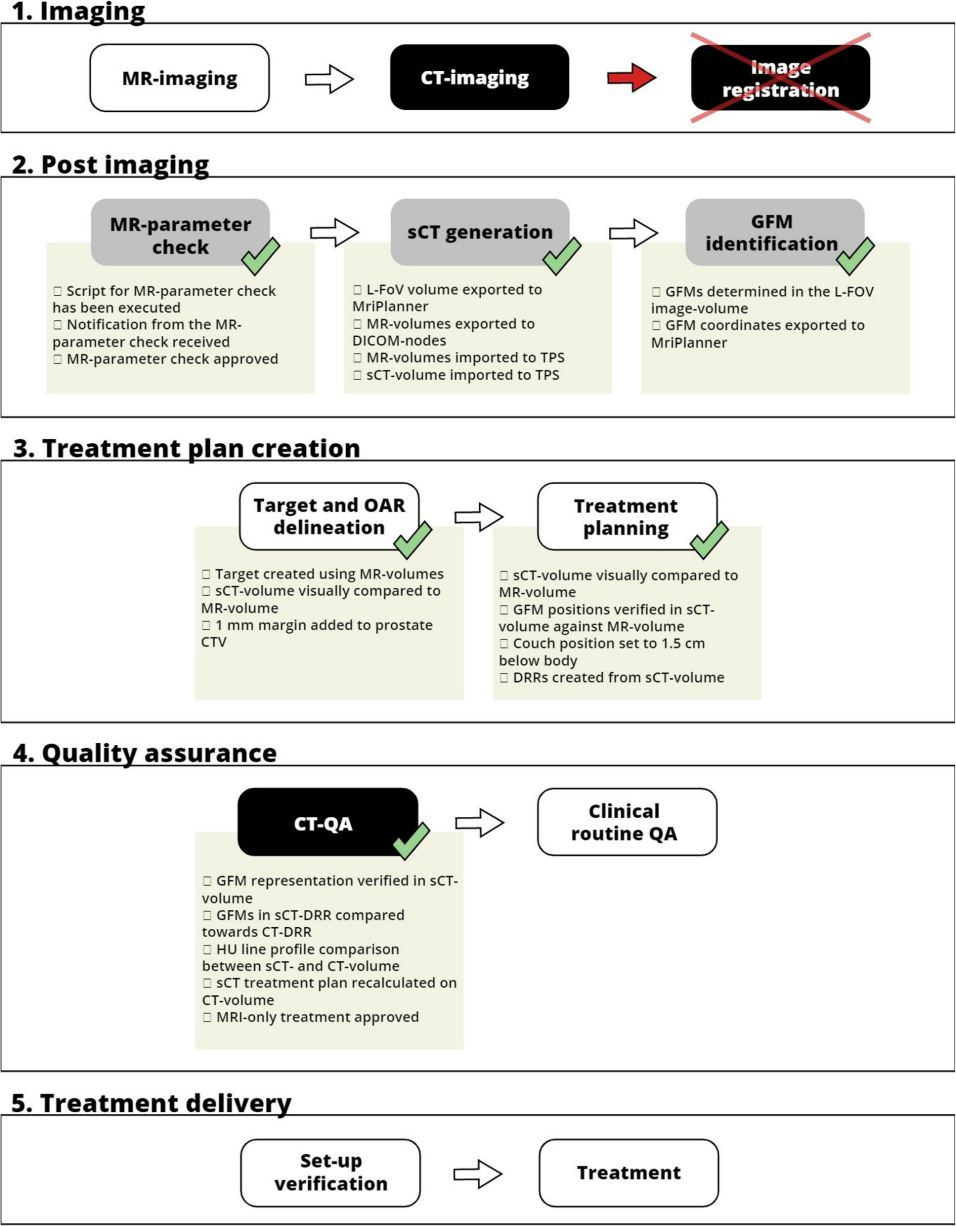
The MR-PROTECT MRI-only study workflow divided into the following five categories: 1. Imaging, 2. Post imaging, 3. Treatment plan creation, 4. Quality assurance and 5. Treatment delivery. Included patients underwent the workflow from category 1 to 5, following the tasks within each category as indicated by the white arrows. White boxes within each category indicate tasks inherited from the conventional dual-modality workflow. Grey boxes are new tasks specific to MRI-only. Black boxes are tasks incorporated in the study workflow for QA-purposes during the implementation procedure, but will probably not be needed in future clinical routine of MRI-only. No image registration between CT and MR was needed in this MRI-only workflow to facilitate treatment planning. In a future clinical routine MRI-only workflow, the black boxes can be excluded and replaced by appropriate QA-routines not including CT-imaging. Along some tasks in category 1–3, there are green check marks, which indicate the use of an electronic questionnaire. The electronic questionnaire items are shown below each of the corresponding check marks. Definitions: magnetic resonance (MR), computed tomography (CT), large field of view (L-FoV), treatment planning system (TPS), gold fiducial marker (GFM), clinical target volume (CTV), digitally reconstructed radiograph (DRR), Hounsfield Unit (HU), quality assurance (QA)

The MRI-only workflow used in this study was inherited from the dual-modality workflow for prostate cancer patients in our clinic. The goal was to keep the same structure as the previous dual-modality workflow and make adjustments when required for MRI-only. The MR-PROTECT MRI-only workflow is schematically presented in Fig. 1 and its content explained in the figure text.

To enable prospective and retrospective analysis of the workflow, CT-images were acquired for each patient. The CT-images were imported to the clinical systems after planning approval of the MRI-only treatment plan and were not available in the treatment planning system (TPS) during any task prior to the CT-QA. Electronic questionnaires were introduced in the MRI-only workflow using ARIA (v.13.6, Varian Medical systems, Palo Alto, California, USA) to guide the clinical staff during the implementation. If problems were detected during the workflow, a patient could if necessary be converted to the conventional dual-modality workflow. All questionnaire items are found below the respective task (green check marks in Fig. 1). The tasks in the workflow are presented in the following subsections.

### Imaging

#### MR-imaging

The MR-scanner used was a GE Discovery 750 W 3.0 T (Software version DV25.1-R02–1649.a, GE Healthcare, Chicago, Illinois, USA). The MR-QA was performed according to clinical practice during the study, which included a monthly geometric distortion check using a large field of view (FoV) phantom (GRADE, Spectronic Medical AB, Helsingborg, Sweden) [23, 24].

The MR-protocol consisted of a large FoV (L-FoV) T2 weighted (T2w) sequence, primarily used for sCT-generation, target and OAR delineation and GFM definition. Three small FoV (s-FoV) T2w sequences (transverse, coronal and sagittal projections) were acquired for target delineation support and a multi-echo gradient echo (MEGRE) sequence for GFM-identification [25]. The L-FoV images were acquired between the MEGRE and the transversal s-FoV sequence in the sequence order to minimize impact of intra MR-protocol patient motion. To minimize the impact of geometric distortion large receive bandwidth per pixel, 2D together with 3D gradient distortion correction and automatic volume shimming were used. The MR-protocol, sequence parameters and order are presented in Appendix A. Since all tasks were performed primarily in the L-FOV image geometry, no image registration between the different MR-images were needed.

Patients were immobilized with ankle and knee support and scanned on a flat table top using a 16 channel GE GEM Anterior Array coil, positioned on stiff coil bridges. Patient tattoos for user-origin definition and patient RT alignment were created at the MR-scanner. To enable visualization of the tattoos and their locations in the L-FOV MR-images, cone shaped liquid surface markers (Pin Point for image registration 128, Beekley Medical, Bristol, Connecticut, USA) were used.

#### CT-imaging

The CT-imaging was performed directly after the MRI using a Siemens Somatom Definition AS+ (Software version syngo CT VA48A, Siemens Healthineers, Erlangen, Germany) with 3 mm slice thickness and a tube voltage of 120 kV. Patients were positioned as during the MRI, aligned using the tattoos defined at the MR-imaging task. The CT-images were strictly used for QA purposes and retrospective analysis and were imported into the TPS during the CT-QA task.

### Post imaging

#### MR-parameter check

To minimize the risk of an unintentional change in MR-parameters, experienced previously [22], a MATLAB script (v. 2015b, Mathworks Inc., Natick, MA, USA) for MR-parameter check was developed and used. The script compared the MR-parameters in the L-FoV image against a predefined template and notified the user by e-mail regarding compliance to the template or not (source code available at: https://github.com/jamtheim/MRIAcqParameterCheck). Any deviations and corresponding reasons were recorded and further evaluated.

#### sCT-generation

The sCT-generation (MriPlanner™ v.1.1.2, Spectronic Medical AB, Helsingborg, Sweden) has earlier been described and multi-center/multi-vendor validated [22, 26]. The cloud based solution is connected through a DICOM export node on the MR-scanner, facilitating automatic sCT-generation when the L-FoV images are sent to the node. The sCT-images were automatically returned to the TPS (Eclipse v. 13.6, Varian Medical systems, Palo Alto, CA, USA), DICOM-modality-tagged as a CT and placed in the same frame of reference (FoR) as the MR-images.

#### GFM identification

Three cylindrically shaped GFMs (1 mm diameter × 5 mm long) were implanted into the prostate using a biopsy needle 2 weeks prior imaging. The GFMs resulted in signal voids in both the L-FOV and the MEGRE-images. GFMs were identified using the MEGRE-images, which has been described earlier [25], and their spatial positions were manually identified in the L-FoV images. A DICOM-viewer (MicroDicom v.2.7.9, MicroDicom, Sofia, Bulgaria) was used to display the MEGRE-images during GFM-identification. Two operators identified the GFMs in 24 and 11 patients respectively and 5 together. The spatial positions of the GFMs defined as a RT-structure in the L-FoV images were exported to the MriPlanner™ from the TPS, enabling creation of synthetic GFMs in the sCT-images. This required that the GFMs spatial position was defined in a physical slice (and not in between) in the L-FOV images. The synthetic GFMs were represented in one image slice each in the sCT as round high intensity objects with a diameter of 4 mm.

### Treatment plan creation

#### Target and OAR delineation

Delineation of target and OARs was performed based on the L-FoV images, supported by the s-FoV images. A blended view with the different MR-images was used to define the structures in the L-FoV image geometry. The sCT-images were not used for delineation, but the final structures set were created in the sCT geometry (i.e. the L-FoV geometry).

There was no intention to change the MRI-based planning target volume (PTV) compared to the dual-modality workflow. Therefore, based on an earlier study, a 1 mm extra margin (excluding cranio-caudal extension) was added to the clinical target volume (CTV) to compensate for the smaller MRI-based CTV [27]. A 7 mm isotropic margin was used to create the MRI-based PTV according to clinical practice.

#### Treatment planning

A 10 MV volumetric modulated arc therapy (VMAT) treatment plan was created using the sCT for each patient according to clinical practice. Dose prescription was 78 Gy in 39 fractions. The standard HU to ED curve defined in our clinic was used for dose-calculations. The dose-calculations were performed using an Analytical Anisotropic Algorithm (AAA) (v.13.6.23, Varian Medical systems, Palo Alto, California, USA). Minor modifications to the standard treatment planning procedure were needed with respect to the use of a sCT in our TPS. This included a manually inserted and positioned treatment couch structure at a 1.5 cm distance below the sCT body contour, reflecting the thickness of the mattress used during treatment. The treatment user-origin was defined using the liquid markers placed over the tattoos at the MR-imaging task. Digitally reconstructed radiographs (DRR) for patient positioning were created from the sCT at a gantry angel of 0 and 270 degrees.

### Quality assurance

After import to the TPS, the CT-images were automatically rigidly registered based on the bony anatomy in translational directions to the sCT-images. The treatment plan transfer was performed in translational directions only, disregarding any rotations in the registration. Hence, the registration between sCT and CT was also performed in translational directions only. Separate body contours were created for both images. The sCT treatment plan was recalculated on the CT-images using the same field setup and number of monitor units. Deviations between sCT and CT dose-distributions were evaluated based on PTV_mean_ directly in the TPS by transferring the PTV structure to the CT. From experiences in earlier validation studies [22], a PTV_mean_ dose deviation less or equal to 1% for each patient was considered acceptable. Deviations above 1% were further investigated for approval depending on the reasons and magnitude of the deviations. A qualitative comparison of the general appearance of the HU in the sCT- and CT-images was performed using HU-line profiles in the TPS. Verification of the GFMs was performed with visual sanity assessment of the GFMs positions in the sCT- and CT-images and corresponding DRRs. All questionnaire items (Fig. 1) had to be acknowledged to enable MRI-only treatment approval.

After approval of the MRI-only treatment plan, our standard clinical QA was performed including a verification measurement using the Delta^4^ phantom (Scandidos, Uppsala, Sweden) and gamma analysis [28]. The clinical gamma analysis was performed using a 3%/2 mm global criteria and a 15% dose cut-off, with a minimum pass rate of 90% comparing the measured and planned dose.

### Treatment delivery

When all QA-steps were completed, the MRI-only treatment plan was approved and passed on to daily treatment on a TrueBeam accelerator (Varian Medical systems, Palo Alto, California, USA). Patients were positioned with corresponding fixation as during MRI and aligned using the patient tattoos. Set-up verification was performed with daily kilo voltage (kV)-image registration. The synthetic GFMs, represented in the sCT-DRR, were manually registered towards the physical GFMs seen in the orthogonal kV-image pairs. Operators were instructed to match the center of the GFM in the orthogonal kV-images to the center of the corresponding synthetic GFM in the sCT-DRR. From the eleventh patient and forward, the cylindrical GFM shape was added to the sCT as a RT structure around the synthetic GFMs. This was an attempt to facilitate easier detection of prostate and GFM rotations.

### Retrospective investigation

Analyses of the study population were performed to investigate achievable limits of acceptance criteria. This was performed retrospectively and included three tasks in the workflow; 1) GFM identification 2) treatment planning and 3) set-up verification. Each analysis and its method are presented below.

#### GFM identification

The GFM-identification performance was investigated according to the analysis presented by Greer et al. [15] where the common center of mass centroid of all sCT and CT-GFMs were determined and the distances from each GFM to their respective common centroid were calculated. The spatial location of each GFM center of mass (CoM) was determined in the sCT- and CT-images respectively using the method previously described by our research group in section 2.C of the paper by Gustafsson et al. [25]. The absolute distances from each GFM to the common centroid were compared between sCT- and CT-images.

This resulted in three GFM comparisons for each of the 40 included patients, given as a difference in mm, and hence a total of 120 comparisons. Mean GFM deviation for all comparisons was calculated along with the range, root mean square (RMS) and standard deviation (SD). The presence of intra-prostatic calcifications ≥2 mm in the CT-images were measured and noted.

#### Treatment planning

A comparison between the sCT and CT dose-distribution was performed for the DVH-criteria specified in the clinical protocol in our clinic. This included CTV D_min_, PTV D_98_ and D_95_, rectum D_10_, D_15_ and D_30_, Femoral head D_2_ and Bladder D_mean_. Further, D_mean_ was extracted for all targets and OAR. The deviations between the two dose-distributions were calculated as difference in % of the prescribed dose. A gamma analysis was performed using global gamma criteria of 3%/3 mm, 2%/2 mm, 2%/1 mm and 3%/2 mm. A 15% dose cut-off was used and corresponded to the cut-off value used in our clinical gamma analysis. All evaluations were performed using MICE Toolkit (MICE Toolkit™, v.1.0.9, Nonpi Medical, Umeå, Sweden). The translational registration created between sCT- and CT-images in the prospective CT-QA was applied to the CT dose-distribution and was re-sampled to the sCT spatial resolution. The delineated MR-structures were used in the DVH-evaluation of both dose-distributions.

#### Set-up verification

An evaluation of patient set-up using sCT DRRs with synthetic GFMs was performed. The registrations were performed in the Offline Review module in the TPS by one operator. The sCT-DRR and CT-DRR were manually rigidly matched respectively towards orthogonal kV-image pairs acquired during the first three treatments for each patient. This resulted in the registrations sCT-kV and CT-kV. Registrations were performed according to the clinical method where the center of the GFM in the kV-images are manually aligned with the center of the corresponding synthetic GFM in the DRRs.

A CBCT-based bone match strategy was also evaluated. The sCT and CT-images were registered by one operator in the registration module in the TPS using auto-match towards CBCT-images acquired during one fraction for each patient. The auto-match anatomy was defined using a box, including the common bony anatomy in the images. A HU range of 200 to 1700 within the box was used for the match. This resulted in the registrations CT-CBCT and sCT-CBCT. As the clinical set-up verification did not include any rotations in the couch positioning of the patient, the retrospective registration was performed without rotations.

Differences in couch translations in x, y and z directions for the sCT-kV and the CT-kV registrations were compared for the first three treatment fractions for the first nine treated patients. The couch translation differences in x, y and z between the CT-CBCT and sCT-CBCT registrations were compared for all patients.

## Results

Forty patients were included in the study, where 39 patient successfully passed the MRI-only RT workflow. Median age and weight was 72 years [range: 49–81 years] and 86 kg [range: 62–113 kg] respectively. The Gleason score of the tumors ranged between 3 + 4 and 3 + 5 and the mean PSA prior to treatment was 7.7 ng/ml [range: 1.4–24.0 ng/ml]. Three of the patients received androgen deprivation therapy . T stage of the tumors ranged between 1a and 2c. The single patient excluded from the workflow was too large for the 44.8 cm FoV in the L-FoV sequence. This was detected after the MR-scan was completed and caused lack of signal in the peripheral parts of the body contour. The treatment workflow was successfully converted to the dual-modality CT/MR workflow according to protocol.

The system specific geometric distortion of the MR-scanner were found to be stable over a 15 months period, during which patient inclusion was ongoing (Table 1).

**Table 1:** Worst and mean observed distortion measured from March 2017 to May 2018 on the MR-scanner

Geometric distortion over 15 months (mm)				
Radial distance from isocenter	< 100	100–150	150–200	200–250
Mean distortion (1 SD) [range]	0.2 (0.0) [0.1–0.2]	0.3 (0.0) [0.2–0.4]	0.5 (0.1) [0.4–0.7]	1.9 (0.1) [1.7–2.0]
Mean of max distortion (1 SD) [range]	0.6 (0.1) [0.4–0.7]	0.8 (0.2) [0.6–1.1]	1.6 (0.3) [1.4–2.6]	7.9 (0.3) [7.4–8.6]

For 13 patients, the MR-parameters were not as specified by the template in the MR-parameter check (Table 2). None of the deviations from the template were found to have a clinical impact, which was confirmed using the prospective CT-QA.

**Table 2:** MR-parameter check deviations during the study

Deviation in MR-image parameter	Number of patients	Comment
Extended FoV	7	Used FoV intendedly defined > 44.8 cm. Accepted solution for larger patients
Number of image slices not as specified	3	Specific absorption rate (SAR) limitation transcended, due to low patient body weight
MR-sequence protocol order	2	Order of MRI-sequences in protocol were changed by operator mistakes
Script error	1	MR-parameter template non-compliance due to deviations in sequence-prescanning options

**Fig. 2:**
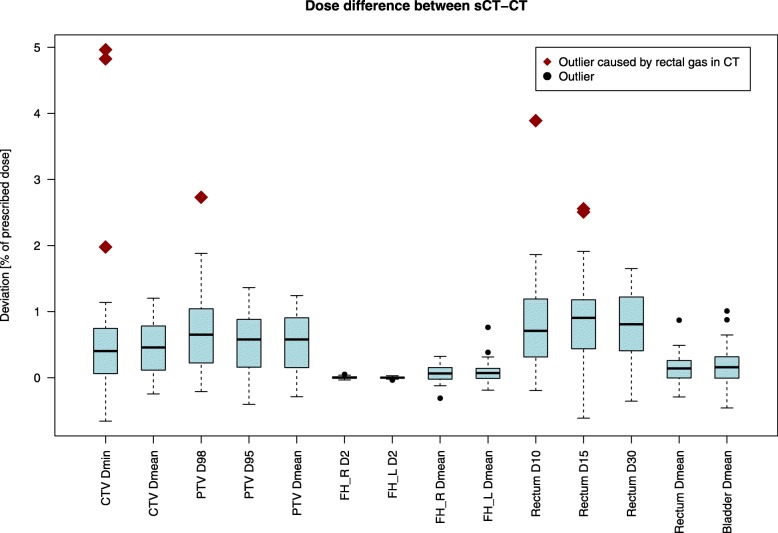
Comparison of target and OAR-doses for sCT and CT dose-distributions. Deviation showed in % of the prescribed dose of 78 Gy for the DVH-parameters in the clinical protocol used for treatment planning. Outliers are represented as black circles. Three patients in the study cohort had dose deviations between sCT and CT that exceeded 2% in CTV_min_, PTV_D98_, Rectum D_10_ or Rectum D_15_ seen as red diamonds. The remaining population had deviations below 2% for all DVH-parameters. The explanation to the deviations above 2% was rectal gas in close connection to the CTV in the CT-images. This was concluded by replacing the rectal gas for these three patients with HU = 0 in the TPS and recalculation of the plan. All deviations were after recalculation below 2% in comparison to the sCT dose-distribution and the outliers were eliminated

**Fig. 3:**
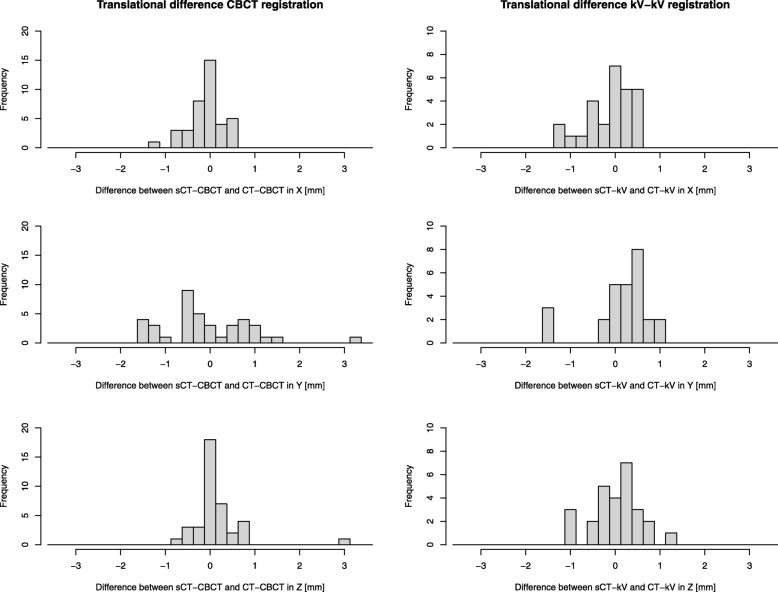
Difference between sCT-CBCT and CT-CBCTregistrations (left column) and CT-kV and sCT-kV registrations (right column) are shown for the three translational directions, x (first row), y (second row) and z (third row). Used bin size were 0.25 mm

The mean difference in marker distances to the respective centroid in sCT and CT was 0.1 mm ± 0.6 mm (1 SD) [range: − 1.1 – 2.2 mm]. The corresponding RMS deviation value was 0.6 mm. The largest range in deviations were seen in the slice direction where mean difference was 0.0 mm ± 1.0 mm (1 SD) [range: − 2.7 – 2.7 mm]. Intra-prostatic calcifications were found in 22/40 (55%) patients. DVH-analysis, gamma-evaluation and set-up verification results are presented for all patients in Fig. 2, Table 3, and Fig. 3.

**Table 3:** Global gamma pass-rates for comparison between sCT and CT dose-distributions using a 15% dose cut-off

Gamma criteria	Gamma pass rate ± 1 SD [range] (%)
3%/3 mm	99.8 ± 0.2 [99.2–100]
3%/2 mm	99.7 ± 0.3 [98.9–100]
2%/2 mm	99.7 ± 0.3 [98.7–100]
2%/1 mm	99.1 ± 0.4 [98.0–99.7]

## Discussion

In the present study MRI-only treatment could be delivered to 39 out of 40 included patients following the study workflow. The study shows the feasibility of an MRI-only workflow, prospectively validated using CT-QA, incorporating a multi-vendor compatible [22] sCT generation method. Further, an image registration free strategy was shown successful, with all final decisions made in a single MR-image geometry. The excluded patient was too obese for the pre-defined 44.8 cm left-right FoV of the L-FoV sequence. This finding made us decide to extend the FoV for larger patients, and demonstrates the value of a prospective feasibility set-up during implementation. A total of seven patients were imaged with an extended FoV and their CT-QA were in accordance to the remaining study population. This adaption could not have been safely performed without the prospective CT-QA.

All workflow tasks, including imaging, post imaging tasks, treatment plan creation, QA and treatment delivery, was completed according to the study workflow for all included patients. Any change in MR-sequence parameters was effectively notified to the user by e-mail by the MR-parameter check. Thirteen deviations were found, but none had any clinical impact. GFMs positions were determined in the L-FoV image for all patients and the GFM locations were confirmed using the prospective CT-QA. Maximum difference in marker distance to the respective centroid in sCT and CT was 2.2 mm. The DVH-analysis showed maximum dose deviations between sCT and CT dose-distributions below 2% of the prescribed dose for all investigated DVH-parameters when the outliers caused by rectal air were corrected for. The gamma pass rates were above 98% for all patients and criteria.

The success-rate for the MR-PROTECT MRI-only workflow was 97.5% (39/40), compared to 100% reported by Greer et al. [15], 92% (184/200) by Tenhunen et al. [14] and 87.5% (42/48) by Tyagi et al. [17] in their corresponding treatment studies. There has been two other studies reporting MRI-only treatments, where one study demonstrated a single treatment [16] and the other did not report success-rate [18]. Tenhunen et al. and Tyagi et al. allowed inclusion of patients with hip-implants while the present study and the study by Greer et al. did not. Avoidance of patient with hip-implants in the inclusion seem to increase the chance of successful MRI-only treatment. Patient obesity, which was the single reason for exclusion in the present study, has also been reported a problem using the MRCAT [14, 17]. Recently, treatment planning using MriPlanner™ sCTs for patients with hip-implants were investigated [29]. In this study, using a VMAT prosthesis-avoidance planning approach, dose differences were in the range of − 1.0-0.9% for PTV and OARs mean doses. The study was performed using a 1.5 T MR-scanner, without modification to the non-hip-implant training atlas. In clinical practice of MRI-only, there will be examples of patients which cannot receive MRI-only treatments. This can for example be patients with obesity, metallic-implants or other MR-contraindications. Unfortunately, this introduce a treatment practice inhomogeneity for prostate cancer patients when MRI-only are used. Since metallic implants in the pelvic area for the prostate cancer cohort are common, this area has to be further explored to enable treatment for a wider range of patients.

From the eleventh patient and forward in our study, the complete GFMs were delineated in the TPS and included the sCT DRR. This radiotherapy structure enabled easier detection of prostate and GFM rotations. The fiducial marker identification was restricted to be performed in one physical slice (and not in between) in our method. This was a prerequisite for synthetic GFM representation in the sCT by the vendor. The highest deviation in GFM to centroid distance of 2.2 mm presented is just below the 2.5 mm slice thickness of the L-FOV image. The restriction in slice-thickness is a probable reason for the higher deviation compared to the 1 mm limit used by Greer et al. [15]. Contrary to a previous published study [17], GFM-identification, target and OAR delineation required no image registrations and was performed completely without the use of CT in our study. Several studies have also explored automatic GFM-identification [21, 25, 30] which could be an alternative to manual identification. However, until these methods have reached 100% detection accuracy as the manual identification method used in this trial, automatic methods should only be used as an aiding support tool to minimize observer bias and speed up the manual GFM-identification.

The dose deviations showed in this study are low and gamma pass rates high and in good agreement with the results presented in the previous multi-center treatment planning validation study MR-OPERA [22]. All dose-comparisons are also within the earlier proposed criteria for reliable use of MRI-only of 2% [31]. Air pockets in the vicinity of the CTV should be noted and tentatively replaced with appropriate HU in the CT-images to avoid discrepancies from the sCT-images. Unintendedly and unnoticed change in MR-parameters, seen in the MR-OPERA study, were in this study monitored with automatic MR-parameter check. Experience and training of the MR-staff can be an explanation to the absence of unintentional changes in the present study. In the FEMA by Kim et al. [19], education along with questionnaires and automatic checks were suggested as risk mitigations, which in our study were proven to be effective tools. A solution could also be to have a fixed MR-sequence used for sCT-generation, without possibility to change the MR-parameters.

To facilitate future implementations of MRI-only, and aid widespread implementation, guidelines are of highest importance and has recently been suggested [32]. In such guidelines, limits of acceptance criteria should be suggested. With the limited number of studies using acceptance criteria, this has not been possible to establish so far. According to our results, acceptance criteria of 2% dose deviation between sCT and CT dose-distributions could appropriately be applied by future clinical implementations, as earlier suggested [29]. The previous suggested gamma analysis acceptance criteria of 90% was easily reached in the present study, in which a gamma pass rate above 98% was achievable for all patients. Acceptance criteria for GFM-identification are dependent on the slice thickness of the MR-images and method used for GFM-identification and representation in the sCT. The limit of the acceptance criteria should thereby be adjusted depending on the used method. If the GFM-identification are not restricted to a physical slice, a narrower limit than 2.5 mm could be achievable. MRI-only based patient positioning with both CBCT and DRRs has been presented earlier for other sCT methods [32]. Our results indicate that patient positioning using MriPlanner can be performed within a 2 mm deviation from a CT-based positioning strategies for most patients.

After successful implementation of MRI-only, in a clinical routine MRI-only workflow, QA-routines to compensate for the missing CT-information are needed. The GFM-identification process can be verified using for instance X-ray images acquired during GFM-implantation [33]. MRI-only GFM- identification which earlier has been reported a problem in MRI-only [14, 17] may thereby be managed using MEGRE. Even though sCT dose-calculation has been widely investigated, there still seems to be a need for QA to detect large dosimetric errors. In clinical routine, the sCT error detection could be performed directly after the first fraction using CBCT [34].

## Conclusions

The MR-PROTECT trial demonstrates the feasibility of a new single sequence strategy MRI-only prostate RT workflow with a commercial sCT generation method. Prospective CT-QA was successfully used to ensure a patient-safe implementation of the workflow. We can conclude that the previous acceptance criteria for dose-comparison of 2% dose deviation are suitable. Further, a gamma pass rate of 98% was achieved for all patients. Acceptance criteria for GFM-identification should be set depending on the MR slice thickness and identification method. From the present study, set-up verification was found to be achievable within 2 mm for two common set-up strategies for all but one patient. More prospective workflow investigations of wider range of set-up strategies are however needed to establish appropriate general acceptance criteria. This proposed prospective implementation method was found to be successful and, altogether, creates a foundation for future implementation of MRI-only prostate radiotherapy and thereby exclude CT in the clinical routine.

## Data Availability

The datasets generated during/or analyzed during the current study are not publicly available due to patient privacy concerns and institutional regulations.

## Appendix

**Table 4:** MRI protocol

	T2 Propeller (Tra, Cor and Sag)	Large FOV T2	MEGRE
Sequence order in protocol	Tra 1, Cor 4 and Sag 5	2	3
Primary field of application	Target definition support	sCT-generation GFM-definition Target definition Risk organ definition	GFM-identification
Scan sequence	2D - Periodically Rotated Overlapping Parallel Lines with Enhanced Reconstruction	2D Fast Recovery Fast Spin Echo	2D Multi-Echo Fast Gradient Echo
Slice orientation	Tra, Cor, Sag	Tra	Tra
Frequence encoding direction	–	Right-Left	Anterior-Posterior
Field of view (frequency encoding direction) (cm)	22	44.8	24
Field of view (phase encoding direction) (cm)	22	31.4	24
Matrix size (frequency encoding direction)	352	640	164
Matrix size (phase encoding direction)	352	512	164
Scan pixel size (frequency encoding direction) (mm)	0.63	0.7	1.46
Scan pixel size (phase encoding direction) (mm)	0.63	0.61	1.46
Reconstructed pixel size (frequency encoding direction) (mm)	0.43	0.44	0.47
Reconstructed pixel size (phase encoding direction) (mm)	0.43	0.44	0.47
Bandwidth per pixel (Hz)	473	390	508
Acqusition time	04:43, 04:17, 03:19	07:00	05:36
Slice thickness (mm)	2.8, 3, 3	2.5	2.8
Slice gap (mm)	0	0	0
Repetition time (ms)	9151, 8293, 7381	15,000	1000
Number of slices	32, 29, 23	88	34
Echo time (ms)	96, 96, 109	96	2.38–23.6
Inter-echo time	–	–	3.03 ms
Refocusing flip angle (deg)	120	130	–
Echo train length	28, 28, 32	15	–
Number of averages	2.10, 2, 2	1	2
Number of echoes	1	1	8
Intensity correction	Yes (SCIC)	Yes (SCIC)	Yes (SCIC)
Intensity filter	–	None	None
3D geometry correction	Not available	Yes	Yes
Shimming	Yes (Auto)	Yes (Auto)	Yes (Auto)
Flow compensation direction	–	Slice direction	–
RF transmit mode	Multi transmit	Multi transmit	Multi transmit

## Abbreviations

EBRT: External beam radiotherapy
CT: Computed tomography
MR: Magnetic resonance
ED: Electron density
OAR: Organs at risk
MRI: Magnetic resonance imaging
RT: Radiotherapy
HU: Hounsfield unit
sCT: Synthetic computed tomography
FEMA: Failure mode and effect analysis
QA: Quality assurance
CBCT: Cone beam computed tomography
MR-PROTECT: Magnetic resonance imaging only prostate radiotherapy excluding CT
GFM: Gold fiducial marker
TPS: Treatment planning system
FoV: Field of view
L-FoV: Large field of view
s-FoV: Small field of view
FoR: Frame of reference
CTV: Clinical target volume
PTV: Planning target volume
VMAT: Volumetric modulated arc therapy
DRR: Digitally reconstructed radiograph
kV: Kilo voltage
RMS: Root mean square
SD: Standard deviation
